# Transesophageal echocardiography related complications

**DOI:** 10.3389/fcvm.2024.1410594

**Published:** 2024-06-28

**Authors:** Linyue Zhang, Yuji Xie, Zhaoli Ren, Mingxing Xie

**Affiliations:** ^1^ Department of Ultrasound, Union Hospital, Tongji Medical College, Huazhong University of Science and Technology, Wuhan, China; ^2^ Clinical Research Center for Medical Imaging in Hubei Province, Wuhan, China; ^3^ Hubei Province Key Laboratory of Molecular Imaging, Wuhan, China

**Keywords:** echocardiography, transesophageal, safety, complication, indication, contradiction

## Abstract

Transesophageal Echocardiography (TEE) is an important imaging method for the evaluation of cardiac structure and function, and it holds significant value in the clinical management of cardiovascular diseases. Unlike transthoracic echocardiography (TTE), which is non-invasive, TEE involves semi-invasive intracavity operations, leading to increasing attention to its safety and potential complications. Especially with the increasing demand for TEE applications in clinical practice and the rapid growth in the number of facilities utilizing it, the standardized application and safe operation of TEE technology have become particularly crucial. This article will review the literature and draw upon personal experience to analyze the complications and safety of TEE examinations from a technical perspective.

## 1 Introduction

Transesophageal echocardiography (TEE) represents a revolutionary breakthrough in ultrasound imaging technology. Unlike transthoracic echocardiography (TTE), TEE is not affected by factors such as patient body size, chest deformities, or lung air. It can significantly enhance the resolution of various modalities of cardiac ultrasound images and plays an irreplaceable role in the diagnosis and treatment of various heart diseases where other imaging methods fall short (1, 2). In 1983, Schluter and Henrath first described the clinical application of TEE. Since then, TEE has demonstrated its unique clinical value, leading to rapid development in both technological breakthroughs and application scope (3). Currently, TEE is not only a unique technology for diagnosing various cardiovascular diseases but also an indispensable tool for perioperative management. It serves as the cornerstone of rapidly evolving interventional cardiac treatment techniques (4, 5). Performing a TEE examination involves placing esophageal probes of different sizes and specifications into the esophagus or stomach. In various clinical scenarios, this procedure can lead to complications of varying severity, with severe cases potentially resulting in patient mortality. It has been reported that the incidence of complications associated with TEE ranges from 0.4% to 1.4% (6–9). Freitas-Ferraz et al. and Afzal et al. retrospectively analyzed patients who underwent TEE-guided structural heart disease intervention and found that the incidence of TEE-related complications was 6.1% and 5.3% respectively (1, 10). The safety of TEE requires further understanding and extensive discussion in different patient populations, surgical types, and clinical scenarios.

## 2 Complications related to the TEE probe insertion process

During the TEE examination process, probe insertion is a necessary procedural step. Operators must first have a clear understanding of the indications and contraindications for probe insertion (Table 1), undergo standardized training, and pass assessment before they are permitted to insert the probe. Otherwise, probe insertion may lead to various complications of different severity (Table 2) (11). During the probe insertion process, within the passage from the oral cavity to the esophagus, there are physiological bends between the base of the tongue and the soft palate, and the trachea and esophagus are arranged anteriorly and posteriorly in the pharyngeal region. Additionally, there is a piriform fossa, all of which are anatomical structures prone to causing complications. In particular, improper patient positioning during the procedure, along with angles between the oral cavity, pharynx, and esophagus that are too small, too large, or not aligned horizontally, can lead to difficulties in probe insertion and subsequent complications. Incorrect manipulation techniques or excessive force can also result in complications such as injury to the oral cavity, pharynx, and esophagus.

**Table 1:** Strict vs. relative contra-indications for TEE.

Strict contra-indications	Relative contra-indications
Perforated viscus	History of radiation to neck and mediastinum
Esophageal stricture	History of GI surgery
Esophageal tumor	Recent upper GI bleed
Esophageal perforation, laceration	Barrett's esophagus
Esophageal diverticulum	History of dysphagia
Active upper GI bleed	Restriction of neck mobility
	Symptomatic hiatal hernia
	Esophageal varices
	Coagulopathy, thrombocytopenia
	Active esophagitis
	Active peptic ulcer disease

TEE, transesophageal echocardiography.

**Table 2:** TEE related complications according to severity and corresponding treatment/prevention measures.

	Severity	Prevention	Treatment	Time to resolve
Oropharyngeal				
Lip bruising	+	Use a bite guard and laryngoscope	Apply an ice pack	1–2 days
Lip laceration	++	Lubricate the probe properly	Stitches to close wound	3–5 days
Tooth defect	+	Use a bite block to keep the probe midline	Dental filling or crown	3–5 days
Loose tooth	++	Assess the oral cavity and operate gently	Periodontal therapy/dental replantation	5–7 days
Pharyngeal laceration	++	Place the probe under direct visualization	Restricted oral intake and intravenous antibiotics	3–5 days
Perforation of the hypopharynx	+++	Avoid keeping the probe in a flexed and locked position	Operative suturing or reconstruction surgery	1 month
Accidental tracheal intubation	+++	Place the probe under direct visualization	Refraining from insertion	1–2 days
Esophageal				
Odynophagia	+	Reduce unnecessary operations, freeze image when probe is not being used	Resolve on its own or with medication	3–5 days
Dysphagia	++	Reduce unnecessary operations, freeze image when probe is not being used	Medicine or esophageal dilation	3–5 days
Laceration/perforation	+++	Avoid forceful placement and manipulation of the TEE probe	Operative suturing or reconstruct surgery	3–5 weeks
Mallory-Weiss tear	+++	Refraining from insertion if resistance is met	Endoscopic hemostasis	10 days
Gastric				
Laceration/perforation	++++	Avoid forceful placement and manipulation of the TEE probe	Emergency surgery to prevent acute peritonitis	1–2 months
Hemorrhage	++/+++	Minimize depth manipulation	Observation/stanching medicine with antibotics/ surgery is considered if bleeding is not self-limiting	1 month

### 2.1 Oropharyngeal injury

Complications related to oropharyngeal mechanical injury include tongue and lip pressure injuries, oral mucosal damage, dental injuries, and joint damage.

#### 2.1.1 Tongue and lip pressure injuries

TEE probe can cause pressure on the tongue and lips, leading to swelling and ischemia, but usually, these injuries are mild. Tongue and lip swelling is self-limiting and reversible, often resolving within 1–2 days without treatment. Secondary tongue and lip injuries resulting from TEE probe placement are extremely rare (12). Tongue ulcers occur infrequently. Kallmeyer et al. (12) analyzed complications related to TEE in 7,200 patients undergoing cardiac surgery, with tongue ulcers reported in only 4 cases. Prolonged pressure from the esophageal probe on the mouth or tongue, or when the angle between the probe body and the tongue/lips is too narrow, can lead to stasis of blood flow, resulting in severe ischemia and necrosis of the tongue (13, 14). Therefore, it is essential to pay attention to the overall duration of the TEE examination and the angle between the probe body and the tongue/lips during the procedure.

#### 2.1.2 Oropharyngeal mucosal injury

During the insertion of the TEE probe into the oropharynx, if the tip of the probe does not progress along the midline of the pharynx, it is easy for the probe tip to slip into the left or right piriform fossa. In this situation, if the operator blindly pushes the probe forward or applies excessive force, it can lead to mucosal bruising or even tearing in the oropharynx. The American Society of Echocardiography (15) recommends that during the probe insertion process, the probe should be placed at the midline of the pharynx or the midline of the glottis before being advanced downward along the esophagus to avoid misplacing the probe in the piriform fossa.

#### 2.1.3 Dental injury

Patients with loose teeth, especially those with misaligned dentition, are at risk of tooth dislodgement during TEE examinations. In severe cases, dislodged teeth may even migrate into the trachea or esophagus. The incidence of TEE-related dental injury is approximately 0.03%–0.1% (12, 16). Before TEE examination, it is important to thoroughly inquire about the patient's dental history and carefully examine the oral cavity to help reduce the occurrence of such injuries.

#### 2.1.4 Joint injury

**Figure 1:**
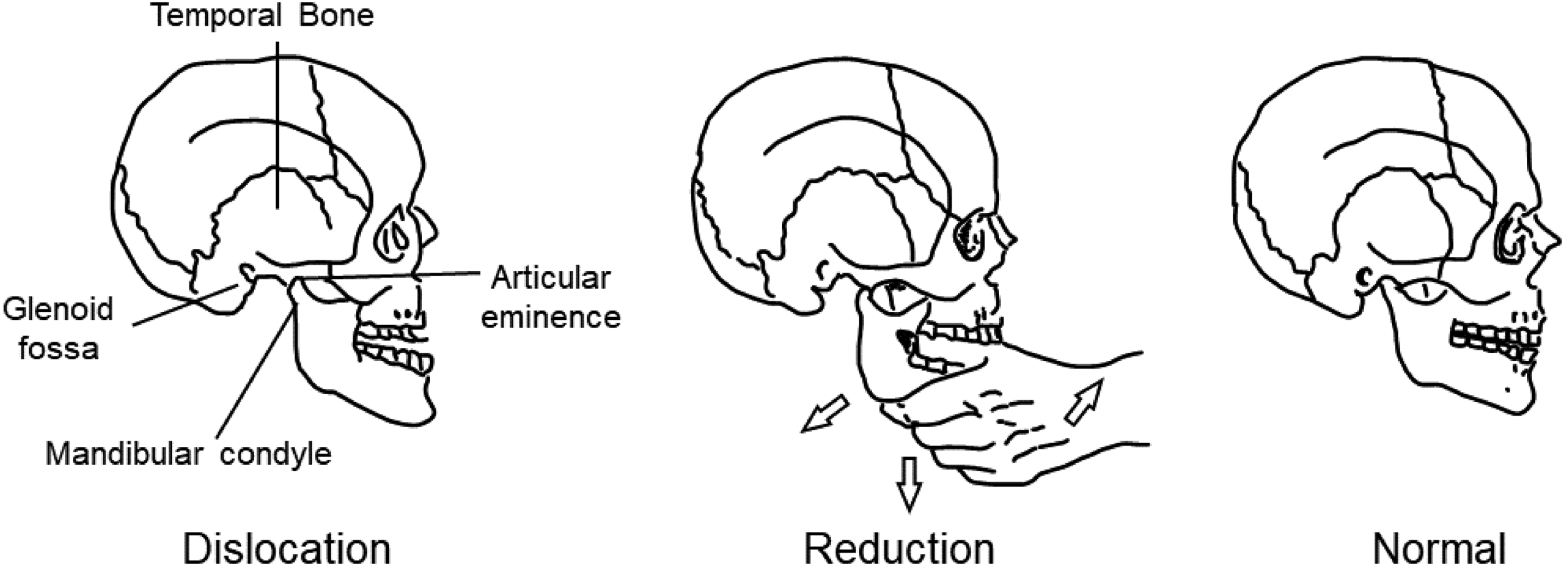
Demonstration on repositioning a temporomandibular joint dislocation.

During the probe insertion process, patients should be instructed to tilt their head backward and raise their chin, ensuring that the oral cavity, pharynx, and esophageal passage remain as straight as possible, facilitating smooth probe insertion. However, excessive force during chin elevation may rarely lead to complications such as temporomandibular joint dislocation (17, 18). If such a dislocation occurs, manual reduction should be performed as soon as possible (Figure 1).

### 2.2 Esophageal injury

During the insertion of the probe, contraction of the pharyngeal muscles can often hinder the advancement of the probe, leading to changes in the curvature of the probe tip. If the curved TEE probe is forcefully advanced or withdrawn in such situations, it can cause mucosal damage, bleeding, and even a series of related complications such as severe esophageal tears or perforations (19).

#### 2.2.1 Esophageal mucosal injury

The severity of esophageal mucosal injury related to TEE varies. Mild cases may involve only slight damage to the esophageal mucosa, while severe cases can result in secondary mucosal tears or ulcers. There are discrepancies in the reported incidence rates of esophageal mucosal injury. In a prospective analysis conducted by Freitas-Ferraz et al. (20) on patients undergoing TEE-guided percutaneous structural heart interventions, the results showed that among 50 patients, 43 individuals (86%) occurred new esophageal hematomas, tears, and abrasions. Both non-anatomical variant occult esophagogastric lesions and anatomical variant occult esophagogastric lesions were identified as risk factors for complications related to TEE probe insertion. Therefore, it is crucial to carefully inquire about the patient's esophageal-related medical history before conducting a TEE examination.

During intraoperative TEE, due to general anesthesia, patients are unable to cooperate with swallowing, and operators must insert the esophageal probe while the patient is unconscious (Table 3). This procedure may lead to mechanical esophageal injury. In contrast, patients under local anesthesia can cooperate with swallowing and respond to TEE procedures, which facilitates safer probe insertion and reduces the risk of TEE-related complications (20). Using auxiliary devices such as laryngoscopes can reduce the difficulty of intubation and the number of insertion attempts, thereby lowering the risk of complications oropharyngeal mucosal injury, esophageal mucosal injury, and postoperative swallowing difficulties (21, 22).

**Table 3:** Pro- and cons of TEE under sedation/general anesthesia vs. awake/general anesthesia.

	Pros	Cons
Sedation/general anesthesia	Patient is unconscious, discomfort or pain is minimized	Hypotension or hypoxia
		Respiratory depression/apnea
	The passage of TEE probe is easier	Potential mechanical esophageal injury
Awake/local anesthetic	Patients can respond to physical stimuli	Short-term memory lapses
	Airway assistance is not required	Hemodynamic compromise

To reduce the occurrence of complications related to TEE probe insertion, operators should be familiar with the anatomical characteristics of the oral cavity, pharynx, and esophagus. To master the correct operating procedure: Firstly, the probe should be smoothly inserted into the mouth in a straight manner. When it reaches the posterior third of the tongue, rotate the handle to curve the probe tip anteriorly and continue advancing, pressing against the base of the tongue. As the probe enters the oropharynx, rotate the handle again to slightly flex the probe tip posteriorly, avoiding the piriform fossa and guiding it into the esophagus. During insertion, gentle pressure should be applied to advance the probe. If significant resistance is encountered, do not force insertion. In most cases, the probe may encounter resistance due to mechanical stimulation, causing contraction of the pharyngeal muscles. In such situations, pause the procedure, ask the patient to relax, or have them swallow a small amount of warm water before resuming the insertion procedure.

#### 2.2.2 Esophageal tear and esophageal perforation

TEE examination-induced esophageal perforation is a rare but serious complication, with an incidence rate ranging from approximately 0.01% to 0.38% (12). Ku et al. (23) reported a case where a patient experienced sudden radiating pain below the sternum, accompanied by profuse sweating, nausea, and vomiting 6 h after a TEE examination. Enhanced CT scans and esophagography confirmed esophageal perforation. Emergency esophageal repair surgery was performed. Sainathan et al. (24) reported that the majority of intraoperative TEE-related esophageal perforations occurred in the thoracic and abdominal segments of the esophagus, with incidence rates of 72% (18/26) and 16% (4/26), respectively. For non-intraoperative TEE examinations, patients are typically under conscious sedation. Due to intact pharyngeal reflexes, esophageal perforations during TEE probe insertion primarily occurred in the cervical and pharyngeal regions, with incidence rates of 55.6% (5/9) and 33.3% (3/9), respectively. The risk factors associated with esophageal perforation include esophageal strictures, Zenker's diverticulum, radiation-induced esophageal fibrosis, drug-induced esophageal ulcers, and esophageal compression caused by severe cardiac enlargement (25, 26). These factors can all increase the mechanical pressure exerted by the probe on the esophagus. Due to the insidious and delayed onset of symptoms of esophageal perforation, as well as its low specificity, delayed diagnosis and treatment can further increase the rate of disability and mortality. Freitas-Ferraz et al. (1) conducted the first evaluation of the safety of using TEE in transcatheter structural heart procedures. The results revealed that in high-risk patient groups undergoing procedures such as MitraClip, left atrial appendage closure, and perivalvular leak closure, the overall incidence rate of TEE-related complications was 6.1%, with the esophageal site having a particularly high incidence rate of 1.9%. Royer et al. (27) reported a case of esophageal perforation following TEE in a patient who underwent transcatheter aortic valve implantation due to protruding thoracic vertebrae compressing the esophagus. For patients with cervical or thoracic vertebral osteophytes, careful confirmation of the severity and degree of esophageal compression through other imaging modalities such as CT before TEE procedures is necessary to avoid sharp osteophytes causing frictional injury to the esophagus and leading to tears. Royer et al. suggest that cervical and thoracic vertebral osteophytes should be considered relative contraindications for TEE procedures (27).

## 3 Complications regarding the TEE probes manipulation

Once the probe enters the esophagus through the throat, there is a noticeable decrease in resistance as the probe advances, leading to a significant reduction in patient discomfort. At this point, depending on the diagnostic needs, it may be necessary to position the probe in esophagus or the different parts of stomach, followed by various maneuvers such as rotation, advancement, retraction, and flexion of the probe to obtain diagnostic views or for real-time guidance and monitoring. During imaging procedures, improper manipulation or prolonged mechanical compression can lead to damage and bleeding in the superficially distributed venous area at the gastroesophageal junction (19). Intraoperative TEE requires longer and more frequent probe manipulations, posing a higher risk of complications compared to conventional TEE, especially in interventional surgery for structural heart disease.

### 3.1 Gastrointestinal bleeding

During TEE probe manipulation, mechanical, compressive, or thermal injuries can lead to gastrointestinal bleeding. Patients with anatomic variation or history of gastroesophageal diseases are more likely to experience gastrointestinal bleeding, especially if the image acquisition time is too long. Ramalingam et al. (11) conducted a prospective study of 22,314 patients who underwent TEE in the perioperative period. The results showed that the incidence of perioperative TEE-related complications such as upper gastrointestinal hemorrhage, laceration or perforation was 0.08% and the mortality rate was 0.03%. However, in the case of serious complications such as critical upper gastrointestinal hemorrhage, mortality risk can be up to 40%.

TEE is an integral part of transcatheter percutaneous cardiac interventions and significantly reduces the risk of procedure-related mortality (28). However, in recent years, with the widespread application of TEE in structural heart disease surgeries, the incidence of related complications has gradually increased. Prolonged major surgeries often require continuous manipulation of the TEE probe, which may directly contribute to the increased risk of TEE-related complications (1, 20). In a retrospective study of 12,043 adult patients undergoing structural heart interventions via TEE-guided catheters, Hasnie et al. (29) found that 429 patients (3.6%) experienced severe postoperative complications, with gastrointestinal hemorrhage being the most common. Post-operative gastro-intestinal complications were more common to patients using anticoagulation or antiplatelet therapy or were elderly.

### 3.2 Swallowing dysfunction

Swallowing dysfunction is a major complication following TEE examination, characterized by dysphagia and painful swallowing (6, 20, 30). Actions such as probe insertion, rotation, flexion, and compression can all contribute to postoperative dysphagia. Overheating of the probe due to prolonged work can also cause thermal damage to the laryngeal mucosa, resulting in postoperative difficulty or pain in swallowing for the patient. Plowman et al. (31) performed a standardized assessment of swallowing function in 182 postoperative cardiac patients and showed that TEE image acquisition of more than 100 images was an independent risk factor for patients to experience dysphagia. The frequency of TEE probe manipulation correlates with TEE-related complications. Bolton et al. (32) were the first to use multimodal imaging to evaluate swallowing function in a post-cardiac surgery patient. In this case, continuous pressure from the TEE probe on the pharyngeal wall caused temporary nerve paralysis, resulting in severe subacute swallowing difficulties. Although dysphagia is usually self-limited, complications such as aspiration, airway obstruction, pulmonary infections, or malnutrition can exacerbate symptoms and prolong recovery (33).

## 4 System complications

### 4.1 Cardiovascular system complications

Reports of cardiovascular complications after TEE are rare in general. In a series of 341 obese patients and 323 control patients undergoing TEE, there was only one case of atrial fibrillation (0.29%) and one case of supraventricular tachycardia in each group (0.31%) (35). Another study of 10,419 patients only found three cases of non-sustained ventricular tachycardia (0.03%), three cases of transient atrial fibrillation (0.03%), and one case of third-degree atrioventricular block (0.01%) (34). As for the pathophysiology of TEE induced arrhythmias, the mechanism remains to be elucidated. What acts as triggers for arrythmias is debatable. Some argue the release of adrenergic hormones is the initiating agent (19). Others believe hypoxemia and hypercarbia from procedural sedation may also play a part in this setting. Since the lower esophageal sphincter is innervated by vagus nerve (36). We speculate esophageal stimulation may trigger vagal response, thus inducing arrythmias. Removing the TEE probe and reducing stimulation to the esophagus usually terminates arrhythmias. If symptoms persist, the examination should be terminated immediately, and antiarrhythmic and defibrillation treatments should be initiated.

### 4.2 Respiratory complications

TEE maneuvers also lead to tracheal complications, including tracheal compression, endotracheal tube misplacement (37), aspiration and accidental tracheal intubation. Children are more susceptible to TEE-related tracheal complications than adults due to the smaller internal diameter of the trachea (38). Davies et al. (37) reported the first case in which the kinked tracheal tube caused by a TEE maneuver in turn led to severe airway obstruction in a patient. Michelet al. (39) conducted a retrospective study of 424 pediatric patients undergoing congenital heart surgery and extracorporeal circulation and analyzed the need for reintubation due to upper airway obstruction within 12 h of extubation. The results showed a correlation between intraoperative TEE use and extubation failure, which may be related to vocal cord injury and airway swelling caused by TEE probe insertion and manipulation. Plowman et al. (31) performed a standardized fiberoptic endoscopic swallowing assessment in 182 adult patients who underwent cardiac surgery within 72 h of post-extubation, and this prospective study demonstrated that TEE image acquisition of more than 110 images during the procedure was an independent risk factor for the occurrence of aspiration in patients after cardiac surgery. The incidence of accidental tracheal intubation during TEE has been reported in four out of 1,500 examinations (0.27%) in ambulatory adults (40). According to literature reports, such occurrences may be identified by symptoms of stridor and incessant cough (40). However, these symptoms may not be prominent because of disease and sedative medication. Tracheal probe placement should be suspected in instances of poor image quality, resistance to probe passage, absence of visualizations of the short axis of the aortic valve and pulmonary artery bifurcation at 30 cm from the incisors and appearance of long-axis perspectives of the aortic arch similar to those attainable from a suprasternal transducer position (41, 42).

## 5 Future directions

TEE is a relatively safe imaging technology, but it still requires a high degree of attention to its associated complications. With the increasing demand for TEE in clinical applications and its expanding scope, especially in the context of rapid development in cardiac interventional ultrasound, the types and incidence rates of TEE-related complications vary significantly from the past. Therefore, it is crucial to correctly understand the potential complications in different clinical scenarios. The establishment of TEE training standards helps improve the proficiency of operators and ensures the standardized application and safe operation of TEE. In the event of unexpected TEE complications, corresponding contingency mechanisms should be established to minimize harm to patients as much as possible.
